# Effects of a strategy for the promotion of physical activity in students from Bogotá

**DOI:** 10.11606/S1518-8787.2018052017173

**Published:** 2018-07-13

**Authors:** Leidys Gutiérrez-Martínez, Rocío Gámez Martínez, Silvia A González, Manuel A Bolívar, Omaira Valencia Estupiñan, Olga L Sarmiento

**Affiliations:** I Universidad de los Andes. Facultad de Medicina. Grupo de Epidemiología. Bogotá, Colombia; II Fundación Valle del Lili. Centro de Investigaciones Clínicas. Cali, Colombia; III Instituto Distrital de Recreación y Deporte. Bogotá, Colombia; IV Children’s Hospital of Eastern Ontario Research Institute. Healthy Active Living and Obesity Research Group. Ontario, Canada; V Universidad de los Andes. Facultad de Ingeniería. Centro para la Optimización y la Probabilidad Aplicada. Bogotá, Colombia

**Keywords:** School Health, Exercise, Mobile Applications, utilization, Body Mass Index, Sedentary Lifestyle, Health Behavior, Health Promotion, Salud Escolar, Ejercicio, Aplicaciones Móviles, utilización, Índice de Masa Corporal, Estilo de Vida Sedentario, Conductas Saludables, Promoción de la Salud

## Abstract

**OBJECTIVE:**

To examine the effect of the promotion of physical activity during recess on the levels of physical activity, sedentary behaviors, and adiposity of Colombian students.

**METHODS:**

Three schools were randomly selected by an intervention group in Bogotá, Colombia, in 2013: Intervention (Active Module of Active Recess – MARA) + Text Messages (SMS) (MARA+SMS group), intervention (MARA group), control (control group). Intervention was implemented for ten weeks. The duration and intensity of physical activity and sedentary behaviors were measured objectively using accelerometers Actigraph-GT3X+. Adiposity was measured by body mass index and fat percentage. We measured at baseline (T0) and during the tenth week of intervention (T1). We evaluated the effect of the intervention using a difference-in-difference analysis (DID).

**RESULTS:**

We included 120 students (57.5% girls; mean age = 10.5 years; standard deviation [SD] = 0.64). There was a significant increase in the mean daily minutes of moderate to vigorous physical activity in the MARA group (Difference T1-T0 = 6.1 minutes, standard error [SE] = 3.49, p = 0.005) in relation to the control group. There were no significant changes in the minutes of moderate to vigorous physical activity in the MARA+SMS group (Difference T1-T0 = -1.0 minute; SE = 3.06; p = 0.363). The minutes decreased in the control group (Difference T1-T0 = -7.7 minutes; SE = 3.15; p = 0.011). The minutes of sedentary behaviors decreased in the MARA and MARA+SMS groups and increased in the control group (MARA Difference T1-T0 = -15.8 minutes; SE = 10.05; p= 0.279; MARA+SMS Difference T1-T0 = -11.5 minutes; SE = 8.80; p= 0.869; Control Difference T1-T0 = 10.9 minutes; SE = 9.07; p = 0.407). There was a higher participation in the MARA group in relation to the MARA+SMS group (MARA group = 34.4%; MARA+SMS group = 12.1%). There were no significant changes in adiposity at 10 weeks according to difference-in-differences analysis (body mass index p: ΔMARA+SMS group *versus* Δcontrol group = 0.945, ΔMARA group *versus* Δcontrol group = 0.847, ΔMARA+SMS group *versus* ΔMARA group = 0.990; FP p ΔMARA+SMS group *versus* Δcontrol group = 0.788, ΔMARA group *versus* Δcontrol group = 0.915, ΔMARA+SMS group *versus* ΔMARA group = 0.975).

**CONCLUSIONS:**

The Active Module of Active Recess is a promising strategy to increase physical activity levels and decrease sedentary behavior in students. The addition of Text Messages was not associated with increased moderate to vigorous physical activity or changes in adiposity.

## RESUMEN

**OBJETIVO:**

Examinar el efecto de una intervención de promoción de actividad física durante el recreo en los niveles de actividad física, comportamientos sedentarios y adiposidad de los escolares de Colombia.

**MÉTODOS:**

Tres colegios fueron asignados aleatoriamente a un grupo de intervención en Bogotá, Colombia en el 2013: Intervención (Módulo Activo Recreo Activo – MARA)+Mensajes de Texto (SMS) (grupo MARA+SMS), intervención (grupo MARA), control (grupo de control). La intervención se implementó durante 10 semanas. La duración e intensidad de actividad física y comportamientos sedentarios se midieron objetivamente usando acelerómetros Actigraph-GT3X+. La adiposidad se midió mediante índice de masa corporal y porcentaje de grasa. Se realizaron mediciones en línea de base (T0) y durante la décima semana de intervención (T1). El efecto de la intervención se evaluó utilizando un análisis de diferencia-en-diferencias (ADD).

**RESULTADOS:**

Fueron incluidos 120 escolares (57,5% niñas; edad promedio = 10,5 años; desviación estándar [DE] = 0,64). Hubo un aumento significativo en el promedio diario de minutos de actividad física moderada-vigorosa en el grupo MARA (Diferencia T1-T0 = 6,1 minutos; error estándar [EE] = 3,49; p = 0,005) con respecto al grupo de control. No se observaron cambios significativos en los minutos de actividad física moderada-vigorosa en el grupo MARA+SMS (Diferencia T1-T0 = -1,0 minuto; EE = 3,06; p = 0,363). En el grupo de control los minutos disminuyeron (Diferencia T1-T0 = -7,7 minutos; EE = 3,15; p = 0,011). Los minutos de comportamientos sedentarios disminuyeron en los grupos MARA y MARA+SMS y aumentaron en el grupo de control (MARA Diferencia T1-T0 = -15,8 minutos; EE = 10,05; p = 0,279; MARA+SMS Diferencia T1-T0 =-11,5 minutos; EE = 8,80; p = 0,869; Control Diferencia T1-T0 = 10,9 minutos; EE = 9,07; p = 0,407). Se registró mayor participación en el grupo MARA respecto al grupo MARA+SMS (grupo MARA = 34,4%; grupo MARA+SMS = 12,1%). No hubo cambios significativos en adiposidad en las diez semanas según análisis de diferencia-en-diferencias (índice de masa corporal p: Δgrupo MARA+SMS *versus* Δgrupo de control = 0,945, Δgrupo MARA *versus* Δgrupo control = 0,847, Δgrupo MARA+SMS *versus* Δgrupo MARA = 0,990; PG p Δgrupo MARA+SMS *versus* Δgrupo control = 0,788, Δgrupo MARA *versus* Δgrupo control = 0,915, Δgrupo MARA+SMS *versus* Δgrupo MARA = 0,975).

**CONCLUSIONES:**

El Módulo Activo Recreo Activo es una estrategia prometedora para incrementar los niveles de actividad física y disminuir comportamientos sedentarios en escolares. Adicionar Mensajes de Texto no se asoció con incremento de actividad física moderada-vigorosa o en cambios en adiposidad.

## INTRODUCTION

Childhood overweight and obesity are a global public health problem^1^ and at least 10% of the children in the world are overweight or obese^2^. Between 22.5 and 25.9 million children suffer from overweight or obesity in Latin America^3^, and this number reaches one in six children and adolescents aged between five and seventeen years in Colombia^4^. The promotion of physical activity (PA) and the decrease of the time dedicated to sedentary behaviors (SB) are fundamental in preventing childhood obesity^5^ ^,^ ^6^. However, 74% of the population aged between 13 and 17 years do not follow the recommendations of PA and 57.9% of the children aged between five and 12 years dedicate two hours per day or more to SB in Colombia^4^ ^,^ ^7^ ^,^ ^8^.

Physical education classes and recess time are fundamental for the promotion of PA in students, since children and adolescents spend between six and eight hours a day in school^9^. Consequently, strategies involving recess time are promising interventions for the promotion of PA in students^10–13^.

The incorporation of information technologies has shown the potential to promote healthy lifestyles and health-related structural changes, such as text messages (SMS) that encourage healthy behaviors through immediate feedback and advice^14^ ^,^ ^15^.

Policies exist to extend the student journey in Colombia, strengthening areas such as mathematics, natural sciences, and English, but they do not include PA or sports activities^16^. However, Bogota was a model for the promotion of PA in students through district programs aimed at the school community^17^. Successful local experiences of community programs in urban settings have been described^18^ ^,^ ^19^, but studies on the impact of interventions together with SMS to promote PA and decrease SB in student settings are limited. Therefore, the objective of this study was to examine the effect of a intervention for the promotion of PA during recess, intensified by a SMS strategy, on the levels of PA, SB, and adiposity in students from Bogotá.

## METHODS

This is a randomized community trial carried out with fifth grade students from three schools located in San Cristóbal in Bogotá, DC, Colombia, between July and November 2013.

The included schools were selected from the 20 eligible schools of the International Study of Childhood Obesity, Lifestyles, and the Environment (ISCOLE)^20^. We performed a previous random sampling with the dimensions of socioeconomic level (1, 2, and 3), number of students (1,200–3,400), level of self-reported PA in a previous study (4.9%–11.8% of compliance with the recommendations of moderate to vigorous physical activity [MVPA]), and distance from the school to the Ciclovia (< 1 km). The Ciclovia is a program which temporarily closes the streets to all motorized transport allowing only the access to persons for recreational activities^21^. The number of included schools was determined by the availability of resources for the new implementation of the Active Module of Active Recess (MARA) by the District Institute of Recreation and Sport (IDRD).

The intervention was part of the multi-component program named *Movam Se Estudantes* of the IDRD of Bogotá. This strategy seeks to promote PA in students by integrating elements, structured activities, and supervision. The activities were designed to improve cardiovascular capacity, speed, muscle strength, coordination, and teamwork skills^21^.

The MARA module transformed the usual recess space with standardized activities lasting 20 minutes from the 30 minutes of the usual recess time, three times a week for 10 weeks. The MARA module included 30 PA sessions that combined games with elements such as bows, balls, hoops, ladders, parachutes, tablecloths, rumba, and gaming sessions. In case of unforeseen circumstances to carry out the programmed activity, an alternative was used. All sessions were directed and supervised by the PA managers of the IDRD^a^.

The MARA+SMS group received a daily SMS to promote the participation of the students in the intervention and its dissemination among partners, to motivate the performance of the PA in the context outside school and with the family, and to encourage a healthy diet. The SMS written by expert psychologists were sent to the cell phones of parents and some students, according to the approval of parents and the instruction of the Ethics Committee of the Universidade dos Andes. Parents were asked to show their children every SMS received, if the participant did not receive it directly.

**Figure 1:**
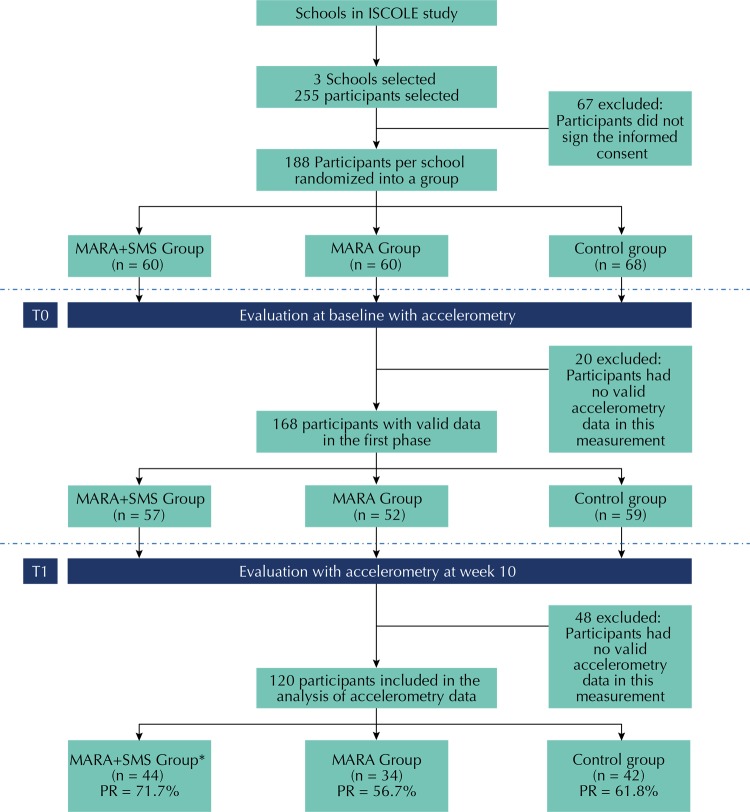
Flowchart of the sampling.

ISCOLE: International Study of Childhood Obesity, Lifestyles, and the Environment; MARA+SMS group: Intervention of Active Module of Active Recess + Text Messages; MARA group: Intervention of Active Module of Active Recess; BMI: body mass index; T0: baseline at time zero; T1: follow-up period at week 10 of intervention; PR: percentage of response

The three selected schools were randomly chosen for an intervention or control group: intervention at recess time (MARA) together with SMS (MARA+SMS group), intervention only (MARA group), and control group (control group) (Figure 1) without any intervention. Data were collected in two periods: before the beginning of the intervention (T0) and during the tenth week of intervention (T1). There was no risk of contamination for the control group as the school was located more than 1 km from the intervention schools, and the implementation of the strategy depended on IDRD personnel.

* One participant in this group dropped out before week 10. However, it was included in the final analysis with the intention-to-treat analysis.

The levels of PA and SB were objectively measured using accelerometers Actigraph GT3X+ (ActiGraph, Pensacola, FL). The accelerometer was used in an elastic belt over the right midaxillary line. Students were instructed to use the accelerometer 24 hours a day for at least seven days, after one day of familiarization, including two weekend days. The minimum acceptable amount of valid accelerometry data included three weekdays, with at least 10 hours of daily use. In the work days, we did not take into account weekend days, since the evaluated intervention was performed on weekdays. The data were compiled with a sampling frequency of 80 Hz, downloaded in one-second epochs and grouped in 15-second epochs for analysis^22^. After data collection, we validated the time of use with software R3.2.3. According to Evenson cut-off points, we defined MVPA as ≥ 574 counts per 15 seconds and physical inactivity as ≤ 25 counts per 15-second epochs^23^. We encouraged the daily use of the accelerometer with decals that the students put on these devices or their journals.

We performed the following anthropometric measurements: size, weight, fat percentage (FP), abdominal perimeter, and the middle third of the arm. Participant size was measured using the portable stadiometer Seca 230 (Hamburg, Germany), using the Frankfort plan. Weight and FP, calculated by bipolar electrical bioimpedance (EBI), were measured using the Portable Body Composition Analyzer Tanita SC-240 (Arlington Heights, IL). Each measurement was taken twice and the mean was used for the analyses. Body mass index (BMI) was calculated using the formula weight (kg)/height^2^ (m^2^). We determined the nutritional status of the children according to BMI using the z-scores derived from the standard deviation (SD) of the measurement, established in the growth patterns of the World Health Organization (WHO)^24^; we grouped risk of thinness and thinness.

The sociodemographic variables included were: sex, age, socioeconomic level, education level, and marital status of the parents. In Bogotá, socioeconomic level is classified from one (low-low) to six (high) according to the characteristics of the households, being 1-2-3 the lowest levels^25^. This information was reported by parents using the sociodemographic and family information questionnaire. Age was calculated with the date of birth and the date of the anthropometric measurements. We evaluated behaviors related to PA, physical inactivity, and eating habits using the diet and lifestyle questionnaire designed for the ISCOLE study and adapted for this study^20^. This questionnaire was administered by a researcher to each participant during intervention days. Some questions related to daily PA and physical inactivity were adapted from the American Youth Risk Behavior Surveillance System (YRBSS)^26^. We designed a component to question the time and type of activities during recess and a satisfaction module about MARA.

Statistical analysis was performed in four phases. First, we carried out a univariate analysis to describe the sociodemographic characteristics, type and frequency of the activities during recess, consumption of food, and anthropometric characteristics. Daily and weekday PA and SB were evaluated as a continuous variable, in mean minutes, with previous log transformation, since the data did not present a normal distribution. Second, we evaluated heterogeneity between groups (MARA+SMS group *versus* MARA group *versus* control group) using Fisher homogeneity test for categorical variables and ANOVA for continuous variables. Third, we performed a bivariate and multivariate analysis with intention-to-treat analysis for students with valid accelerometry data using mixed multilevel models – individual and school – adjusted for sex and age. We evaluated the effect of the intervention using a difference-in-differences analysis (DID) of absolute values, with the models proposed in the previous step, to compare the changes in the levels of PA, SB, and adiposity over time (T0 *versus* T1) among the three study groups. All possible combinations were considered: MARA+SMS group *versus* control group, MARA group *versus* control group, MARA+SMS group *versus* MARA group.

We compared the intensity of some types of activities implemented in MARA by plotting the mean counts per minute of the assistants of a session of each activity in one of the schools involved, using software R3.2.3. Statistical analysis was performed in SAS 9.3 and Stata 13. Data on the continuous PA variables were expressed as means.

The strategy implementation processes were evaluated with eleven semi-structured interviews with the teachers of the participating schools and members of the IDRD team. These interviews were recorded in audio and transcribed verbatim. We took notes when the interviewee did not agree to be recorded. We reviewed the project documents, including protocol, meeting minutes, field work formats, service lists, and SMS. We qualitatively evaluated the process, context, and conditions of the research implementation in these documents. In addition, we analyzed the service lists to understand the dynamics of student participation in the proposed activities.

We sent informed consents to the schools and parents or guardians of potential participants to authorize their inclusion in the study. Similarly, students signed an informed consent accepting their participation. The study was approved by the ethics committee of the Universidade dos Andes (Process 214-2013).

## RESULTS

The total sample consisted of 184 students, and 45.7% complied with the recommendations of PA, according to the mean daily MVPA obtained with accelerometry (MARA+SMS group = 43.9%; MARA group = 31.7%; control group = 59.7%; p = 0.844).

There were no significant differences between groups, except for socioeconomic level. The MARA+SMS group had a higher percentage of boys living in level 3 households (Table 1).

**Table 1:** Characteristics of students from Bogotá by intervention group at baseline in 2013.

Variable	MARA+SMS group		MARA group		Control group		p
		
	(n = 57)		(n = 60)		(n = 67)		
		
	n	%	n	%	n	%	
Sociodemographic							

Sex							
Male	24	42.1	24	40.0	31	46.3	0.789
Female	33	57.9	36	60.0	36	53.7	
Mean age in years and SD^a^	10.4	0.7	10.4	0.6	10.6	0.8	0.125
Socioeconomic level^b^							
1 and 2	37	64.9	58	96.7	63	94.0	< 0.001
3	20	35.1	2	3.3	4	6.0	
Education level of the parents^c^							
Bellow high school – high school	43	75.4	46	76.7	57	85.1	0.346
Licentiate/Vocational school/Undergaduate/Graduate	14	24.6	14	23.3	10	14.9	
Marital status of the parents							
Married	8	14.0	16	26.7	18	26.9	0.160
Divorced/Separated/Widowed	20	35.1	12	20.0	21	31.3	
Never married	29	50.9	32	53.3	28	41.8	

Anthropometric							

BMI z-score for age^d^							
Thin	1	1.8	1	1.7	2	3.0	0.867
Appropriate for age	45	78.9	45	75.0	54	80.6	
Overweight/Obesity	11	19.3	14	23.3	11	16.4	
Fat percentage							
Low	6	10.5	5	8.3	15	22.4	0.111
Normal	43	75.5	46	76.7	43	64.2	
Overweight	4	7.0	4	6.7	8	11.9	
Obesity	4	7.0	5	8.3	1	1.5	
Daily consumption of high-calorie foods							
No consumption	46	80.7	46	76.7	53	79.1	0.876
Daily consumption	11	19.3	14	23.3	14	20.9	
Daily consumption of fruits and vegetables							
No consumption	25	43.9	34	56.7	31	46.3	0.335
Daily consumption	32	56.1	26	43.3	36	53.7	
Mean minutes of daily MVPA^e^	61.9	3.1	56.6	3.5	67.7	3.1	0.141

MARA+SMS Group: intervention of Active Module of Active Recess + text messages; MARA Group: intervention of Active Module of Active Recess; BMI: body mass index

^a^ SD: standard deviation of age in years.

^b^ Socioeconomic level goes from 1 (low-low) to 6 (high) according to the characteristics of the households, and 1-2-3 are the lowest levels.

^c^ The sum of the observations is not equal to total n because of missing values.

^d^ The categories were defined according to the z-scores established in the growth patterns of the World Health Organization (WHO): z < -2 SD thinness; -2 SD < z < -1 DE risk of thinness; -1 SD < z < 1 SD appropriate for age; 1 SD < z < 2 SD overweight; z > 2 SD obesity.

^e^ MVPA: moderate to vigorous physical activity. These results were obtained by accelerometry with n = 120.

The data analysis of the reported AP, accelerometry, and adiposity was performed with 120 students, 36.7% of the MARA+SMS group, 28.3% of the MARA group, and 35.0% of the control group. The control group reported a higher percentage of behaviors associated with PA in relation to the MARA+SMS and MARA groups during most of the recess (p = 0.039) (Table 2).

**Table 2:** Descriptive statistics of the frequency of self-reported activities during recess at baseline and week 10. Bogotá, Colombia, 2013.

Behavior associated with PA	MARA+SMS group				MARA group				Control group				p	
		
	(n = 57)				(n = 60)				(n = 67)					
			
	T0^a^		T1^b^		T0		T1		T0		T1		T0	T1
							
	n	%	n	%	n	(%)	n	(%)	n	(%)	n	%		
Frequency of PA during recess in the last 7 days^c^														
Always	18	31.6	18	31.6	23	38.3	32	53.3	27	40.3	16	23.9	0.737	0.01
Sometimes	35	61.4	36	63.2	31	51.7	26	43.3	33	49.3	47	70.2		
Never	4	7.0	3	5.3	6	10.0	2	3.3	7	10.5	4	6.0		
Most frequent activities during recess in the last 7 days^d^														
Sedentary behaviors^e^	9	15.8	2	3.5	3	5.0	5	8.3	6	9.0	6	9.0	0.039	0.464
Behavior associated with light PA^f^	3	5.3	3	5.3	11	18.3	6	10.0	14	20.9	8	11.9		
Behavior associated with moderate to vigorous PA^g^	45	79.0	52	91.2	46	76.7	49	81.7	47	70.2	53	79.1		

MARA+SMS Group: intervention of Active Module of Active Recess + text messages; MARA Group: intervention of Active Module of Active Recess;

BMI: body mass index; PA: physical activity

^a^ T0: baseline.

^b^ T1: week 10.

^c^ Original question of the questionnaire: How often did you practice a PA during recess in the last seven days?

^d^ Original question of the questionnaire: In the last seven days, which of the following activities were more often during recess?

^e^ This category includes the following activities: eating, sitting (talking, reading, doing school work).

^f^ This category includes the following activities: standing or walking.

^g^ This category includes the following activities: running and playing a little, running and playing some time, running and playing most of the time.

The prevalence of overweight was 19.3% in the MARA+SMS group, 23.3% in the MARA group, and 16.4% in the control group (p = 0.867). The FP was in an optimal range for most students (MARA+SMS group 75.4%; MARA group 76.7%; control group 64.2%; p = 0.111). There were no statistically significant differences between the groups for the indicators of adiposity evaluated neither for the daily consumption of fruits and vegetables or high-calorie food reported (Table 1).

Between baseline and week 10, daily minutes of MVPA were kept in the MARA+SMS group (p = 0.702), increased 6.1 (standard error [SE] = 3.49) minutes in the MARA group (p = 0.044), and decreased 7.7 (SE = 3.15) minutes in the control group (p = 0.005) (DID ΔMARA+SMS group *versus* Δcontrol group; p = 0.005) (DID ΔMARA group *versus* Δcontrol group; p = 0.005). This trend was kept in the mean minutes of MVPA on weekdays.

The daily minutes of SB decreased 11.5 (SE = 8.8) minutes in the MARA+SMS group (p = 0.869) and 15.8 (SE = 10.05) minutes in the MARA group (p = 0.279), and it increased 10.9 (SE = 9.07) minutes in the control group (p = 0.407) (DID ΔMARA+SMS group *versus* Δcontrol group; p = 0.003) (DID ΔMARA group *versus* Δcontrol group; p = 0.003). This trend was kept in the mean minutes of SB on weekdays.

No significant effect was observed on BMI or FP in any of the three groups evaluated (DID BMI p ΔMARA+SMS group *versus* Δcontrol group = 0.945, ΔMARA group *versus* Δcontrol group = 0.847; DID FP p ΔMARA+SMS group *versus* Δcontrol group = 0.788, ΔMARA group *versus* Δcontrol group = 0.915) between baseline and week 10 (Table 3).

**Table 3:** Effect of the intervention on the mean minutes of physical activity, physical inactivity, and indicators of adiposity of students from Bogotá by intervention group in 2013.

Variable	MARA+SMS group		MARA group		Control group		P-values of difference-in-differences analysis		
			
	(n = 44)		(n = 34)		(n = 42)		MARA *versus* Control	MARA+SMS *versus* Control	MARA+SMS *versus* MARA
		
	T0^a^	T1^b^	T0	T1	T0	T1			
Daily MVPA (SE)	61.9 (3.06)	60.9 (3.06)	56.6 (3.49)	62.7 (3.49)	67.7 (3.15)	60.0 (3.15)	0.005	0.005	0.005
MVPA on weekdays (SE)	65.8 (3.27)	64.4 (3.27)	60.2 (3.73)	63.9 (3.73)	72.5 (3.36)	63.3 (3.36)	0.005	0.005	0.005
Daily light PA (SE)	311.9 (7.33)	294.8 (7.33)	279.3 (8.36)	298.0 (8.36)	301.2 (7.54)	281.3 (7.54)	0.002	0.003	0.002
Light PA on weekdays (SE)	318.7 (7.48)	301.1 (7.48)	281.6 (8.54)	305.1 (8.54)	308.9 (7.70)	283.2 (7.70)	0.002	0.003	0.002
Daily physical inactivity (SE)	543.6 (8.80)	532.1 (8.80)	547.6 (10.05)	531.8 (10.05)	502.5 (9.07)	513.3 (9.07)	0.003	0.003	0.001
Physical inactivity on weekdays (SE)	566.3 (9.62)	548.2 (9.62)	561.9 (10.99)	539.9 (10.99)	504.3 (9.92)	523.6 (9.92)	0.003	0.004	0.001
BMI z-score (SE)	0.05 (0.16)	0.1 (0.16)	0.4 (0.19)	0.4 (0.19)	-0.2 (0.17)	-0.1 (0.17)	0.847	0.945	0.990
Fat percentage (SE)	18.9 (0.82)	20.0 (0.83)	20.2 (1.03)	21.1 (1.03)	18.1 (0.93)	18.8 (0.93)	0.915	0.788	0.975

MVPA: moderate to vigorous physical activity; SE: standard error; PA: physical activity; BMI: body mass index

^a^ T0: baseline.

^b^ T1: week 10.

The values of physical activity are the mean minutes.

**Figure 2:**
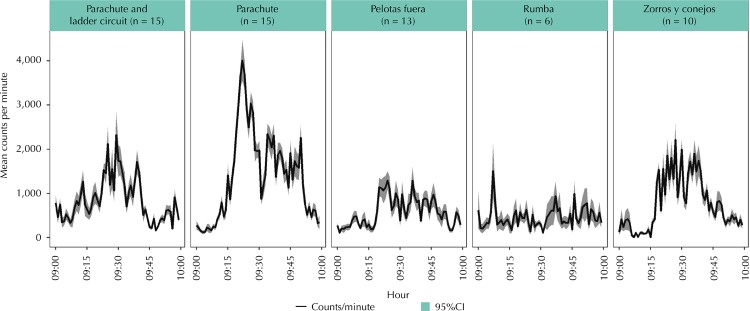
Intensity of the physical activity in the mean counts per minute with 95% confidence interval, measured in one hour of the student’s day* in the participating schools.

Parachute activity reached the highest levels of PA compared to other activities (Figure 2).

* This time of the student’s day includes the 30 minutes of recess in which each activity indicated in the upper part of the Figure was implemented.

Preference was reported for activities that included games with balls (37.1%), games with parachute (21.4%), and rumba (15.7%). Additionally, 47.2% identified MARA as an opportunity to do PA during recess and 94.4% reported interest in keeping doing it.

The environmental, safety, and time factors were the main reasons for no participation in MARA. Approximately 40.0% of the students reported not doing any PA because of an environmental factor (26.6% because of the cold; 12.5% because of the heat). Approximately 10.0% thought it was dangerous to play at recess. Approximately 10.0% of the children reported that recess time and facilities were not enough.

The physical environment, characteristics of the student (ages, preferences, course composition), and type of activity were identified as relevant factors that affected MARA acceptance and participation. The most successful activities identified by the teachers and members of the IDRD were those involving the following elements: parachutes, bows or balls, those related to skills, running, with a lot of movement, and rumba.

Another important factor associated with the implementation and acceptability of the intervention was the work of PA managers in the follow-up of the intervention. Specifically, the constant presence of a team of managers created a safe environment for students, which allowed them to identify with MARA and keep their participation over time. However, the presence of younger students in the MARA was identified as a factor so that the target age group of MARA did not get involved.

Teachers reported that the SMS strategy worked to reinforce the self-esteem of students by their recognition from someone perceived as an external authority figure (the research group). Barriers were identified that might mitigate the effect of the SMS, including difficulties in receiving SMS, lack of interest in MARA or SMS, and perception of an inadequate physical environment.

## DISCUSSION

The main finding of this study is the potential of MARA to increase the minutes of daily MVPA and decrease the minutes of SB in students. The increase of 6.1 minutes in daily MVPA reported in the MARA group represents 10.1% of the recommended daily time for this age group, together with a decrease between 11.5 and 15.8 minutes in SB in the groups with intervention. These results contrast with the decrease in the minutes of MVPA and the increase of SB in the control group. Regardless of the group, there was no effect on the indicators of adiposity.

Our results are consistent with those reported in previous studies on interventions during recess^12^ ^,^ ^13^. Howe et al.^12^ have shown that the participation of students in an intervention with structured recess could contribute with an increase of 8±1.1 minutes of MVPA, that is, an increase from 6.9 (SD = 0.8) to 14.9 (SD = 0.9) minutes in a 30-minute recess. Ridgers et al.^27^ have found that an intervention with redesign of play spaces during recess resulted in an increase of 4.5% in MVPA and 2.3% in vigorous PA during recess. In addition, a systematic review has included nine controlled trials that have evaluated interventions to promote PA during recess. This study has reported an increase in MVPA (between 4.0% and 12.9%) when they were implemented with play elements and marking of areas^28^.

On the other hand, the lack of effect on the indicators of adiposity is debatable in light of the available evidence. The lack of consensus was attributed to the high heterogeneity of duration, intensity, and type of PA of the interventions^29^. A meta-analysis has included 18 studies with interventions in primary school students involving a central component of PA and lasting between 12 and 72 months. This study has exposed the potential of these interventions for a healthy BMI despite the high heterogeneity among the included studies^30^. Therefore, it is possible that the duration of MARA has not been enough to achieve changes in the indicators of adiposity.

The participation of children in PA was associated with entertainment and being appropriate for age^31^. In this study, we obtained a percentage of participation of 34.4% in the MARA group compared to 12.1% in the MARA+SMS group. This suggests an association between participation and increased minutes of PA. According to the satisfaction survey and the process analysis, the perception of disagreement between the age of the students and the proposed activities, as well as the physical environment, negatively affected the participation in MARA. The MARA group had a better physical environment in terms of size and accessibility compared to the MARA+SMS group. This difference could affect the participation in each group, since the students in schools with better physical surroundings got involved up to 20 minutes more in MVPA per week^32^ ^,^ ^33^.

The SMS communication strategy was not associated with an increase in the minutes of PA or changes in the indicators of adiposity. In this sense, the success of this strategy is in its ability to reach an emotional level in the person, in addition to communicating a risk^14^. In this study, although 97.7% of the students reported receiving the SMS, they operated in a social context where the decision to participate in MARA was affected by other factors, among them, interest in the activities offered.

This study presented limitations. Only three schools were evaluated, given the limited resources for the new implementation of MARA by the IDRD. Future studies should consider a larger sample. The estimated difference in minutes of PA over the 10 weeks included all students with valid accelerometry data. However, the highest percentage of students participating in MARA with accelerometer was 47.0%. Consequently, the sample evaluated with accelerometry includes students who probably did not participate in the proposed activities. For this reason, the increase in daily minutes of MVPA could not be attributed solely to MARA. However, we can suggested that it has the potential to make students aware of the practice of PA in other settings and times of the day.

The number of MARA sessions was fulfilled according to the methodology exposed, despite the interruptions in its implementation because of the temporary work stoppage in the schools. During the year, there were protests for better working conditions for teachers in the public sector, which led to the interruption of the classes for several days.

The MARA transformed the recess space, adapting to the available infrastructure and incorporating elements of the Colombian culture. However, the interpretation of these results should be made considering the context of a pilot study. Therefore, the number of schools evaluated and the intervention time limit the generalization of the results. Future interventions should consider a previous study of the profile of the students so that the activities offered better fit their interests and implementation should last at least six months. The redesign of the intervention should consider the inclusion of teachers or students in social service of each institution trained by the IDRD.

This is the first time that an objective evaluation of this type of intervention has been carried out in a randomized community trial in Bogotá with the local sports and recreation agency (IDRD). This alliance enabled these results and lessons learned to transcend from the inquiring scope to the practice. The intervention was incorporated with the support of policies in each school and a 40-week PA pedagogical plan was designed in some schools in Bogotá.

In Latin America, student interventions focused on physical education classes were identified as the only community intervention to promote PA with solid evidence for the development of recommendations^34^. This study provides evidence on the effectiveness of MARA to increase PA and decrease daily physical inactivity during recess in the context of a vulnerable sector of the Colombian capital. These findings indicate the potential to escalate a similar intervention to other middle-income countries. In addition, these results could be the focus of a national public policy to protect the active recess of students as a unique opportunity, free of barriers to accessibility, to engage them in more active lifestyles.

## Footnotes

a For the material with the programmed activities, please contact the corresponding author.
